# A new group-based online job interview training program using computer graphics robots for individuals with autism spectrum disorders

**DOI:** 10.3389/fpsyt.2023.1198433

**Published:** 2023-07-03

**Authors:** Yuichiro Yoshikawa, Taro Muramatsu, Kazuki Sakai, Hideyuki Haraguchi, Azusa Kudo, Hiroshi Ishiguro, Masaru Mimura, Hirokazu Kumazaki

**Affiliations:** ^1^Department of Systems Innovation, Graduate School of Engineering Science, Osaka University, Osaka, Japan; ^2^Department of Neuropsychiatry, Keio University School of Medicine, Tokyo, Japan; ^3^National Center of Neurology and Psychiatry, Department of Preventive Intervention for Psychiatric Disorders, National Institute of Mental Health, Tokyo, Japan; ^4^Department of Neuropsychiatry, Graduate School of Biomedical Sciences, Nagasaki University, Nagasaki, Japan; ^5^College of Science and Engineering, Kanazawa University, Ishikawa, Japan

**Keywords:** autism spectrum disorders, job interview, interview skill, robot, computer graphics

## Abstract

**Introduction:**

Job interviews are a major barrier to employment for individuals with autism spectrum disorders (ASD). During the coronavirus pandemic, establishing online job interview training at home was indispensable. However, many hurdles prevent individuals with ASD from concentrating on online job interview training. To facilitate the acquisition of interview skills from home for individuals with ASD, we developed a group interview training program with a virtual conferencing system (GIT-VICS Program) that uses computer graphics (CG) robots.

**Methods:**

This study investigated the feasibility of the GIT-VICS Program in facilitating skill acquisition for face-to-face job interviews in pre-post measures. In the GIT-VICS Program, five participants were grouped and played the roles of interviewees (1), interviewers (2), and human resources (2). They alternately practiced each role in GIT-VICS Program sessions conducted over 8 or 9 days over three consecutive weeks. Before and after the GIT-VICS Program, the participants underwent a mock face-to-face job interview with two experienced human interviewers (MFH) to evaluate its effect.

**Results:**

Fourteen participants completed the trial procedures without experiencing any technological challenges or distress that would have led to the termination of the session. The GIT-VICS Program improved their job interview skills (verbal competence, nonverbal competence, and interview performance).

**Discussion:**

Given the promising results of this study and to draw clear conclusions about the efficacy of CG robots for mock online job interview training, future studies adding appropriate guidance for manner of job interview by experts are needed.

## 1. Introduction

Autism spectrum disorder (ASD) comprises a range of conditions categorized by challenges with social skills, repetitive behaviors, and verbal and nonverbal communication (1). The Centers for Disease Control and Prevention (CDC) in the US estimates that one in 44 children has ASD (2). A previous study (3) estimated that approximately 50,000 youth with ASD turn 18 years old every year in the US. As a necessary component of adult life, employment is one of the most desirable achievements for individuals with ASD when they enter adulthood (4, 5). However, it is challenging for them to obtain or maintain meaningful jobs (6). The Office for National Statistics recently reported that just 22% of adults with ASD have employment of any kind (7).

Job interviews are major barriers to employment for individuals with ASD (8, 9). Many are not good at verbal communication and conveying job-relevant interview content, and they are not confident in their ability to perform job interviews (10). Most importantly, they experience problems with nonverbal communication, which is directly related to poor performance during job interviews (9). Nonverbal communication is as important as verbal communication in job interviews. Certain nonverbal mistakes “(e.g., individuals with ASD not looking the interviewer in the eye, not making adequate facial expressions)” can reduce the chances of getting a job offer, even if the answers to the interview questions are excellent. Moreover, most job interview settings include multiple interviewers, making job interviews more difficult for individuals with ASD.

Applicants are required to practice for in-person job interviews. In the coronavirus pandemic, establishing online job interview training at home is indispensable. However, many hurdles prevent individuals with ASD from concentrating on online job interview training. In our previous study, we found that individuals with ASD had higher interpersonal tension during online interview training and lower motivation in an online job interview setting (11).

“The Social Motivation Theory of Autism” (12) suggested that individuals with ASD can be interpreted as extreme cases of reduced social motivation. Social motivation is a powerful force that guides human behavior. It can be described as a set of psychological properties and biological mechanisms that bias individuals to preferentially place themselves in the social world, seek social interactions, feel pleasure, and work to foster and maintain social bonds. Social motivation allows individuals with ASD to establish smooth relationships and promotes coordination. Social communication intervention approaches may be effective in providing motivational activities and settings for individuals with ASD (13).

A previous study using realistic virtual humans reported that individuals with ASD saw agent peers in interview settings using virtual reality while talking less frequently than typical development (14). Our preliminary study confirmed that many individuals with ASD are fearful of realistic virtual humans and avoid looking at them because of complexity of their realism. Unlike humans, virtual humanoid robots operate in predictable and lawful systems and, thus, can provide a highly structured learning environment that allows individuals with ASD to focus on relevant stimuli. Individuals with ASD may be highly motivated to communicate with virtual humanoid robots and exhibit social behaviors toward them (11).

When designing objects for use by individuals with ASD, researchers often subscribe to the idea that “simple is better”; that is, they gravitate toward simple and mechanical objects (15–19). Based on these considerations, the use of a simple virtual agent for training individuals with ASD can be inferred to be appropriate. Studies on virtual exposure using clinical samples have shown that even simple virtual agents can significantly increase anxiety and are more effective for phobics than speaking in front of an empty virtual seminar room (11, 20). In designing a virtual human-robot mock online job interview training for individuals with ASD, it is important to consider how to design the agent’s eyes, because individuals with ASD pay less attention to their eyes than individuals with typical development (21). Increasing eye contact is widely recognized as an important and promising treatment for individuals with ASD (22, 23). To create useful online job interview training that is beneficial to many of these individuals, it is important to the agent in their eyes during training. If individuals with ASD can exercise eye contact with virtual agents, they may overcome their fear of the interviewer’s gaze and may be able to reduce their anxiety during the interview process.

Low motivation for interview training may be partly due to the inability of individuals with ASD to understand others’ perspectives and how they behave in an interview setting. Challenges in this area may arise owing to the impaired ability of individuals with ASD to attribute mental states to themselves and others (24), often called the Theory of Mind (ToM). ToM represents the cognitive ability to infer the mental states of oneself and others and is an essential ability in social cognitive functioning and a core cognitive impairment in individuals with ASD (25). If individuals with ASD cannot put themselves in the position of an interlocutor because of impairments in ToM (24), they are unlikely to understand the impact of their actions on others. Moreover, they may not have been motivated to learn the verbal and nonverbal communication required for successful job interviews. Therefore, interventions from the perspective of ToM are required.

To facilitate at-home interview skill acquisition for individuals with ASD, we developed a group interview training program using a virtual conferencing system (GIT-VICS Program). In this program, five or six individuals with ASD were assigned to a group. Each group comprised one interviewee, two interviewers, and two meta-evaluators. The participants performed all the roles multiple times in random order.

The GIT-VICS Program differs from the method used previously (11) in that (1) the meta-evaluator evaluated the performance of both the interviewee and the interviewers, (2) the virtual interview sessions included two interviewers (compared to only one interviewer in the previous study), and (3) the interviewee was given an additional way to control the gaze of the computer graphics (CG) robot as well as a task to properly present attention to both interviewers. Above all, in this study, to investigate the effectiveness of the GIT-VICS Program, we prepared a face-to-face mock job interview setting (in a previous study, we prepared a mock online job interview setting). The CG robots used in the GIT-VICS Program show a range of simplified expressions that are simpler and less complex than real human faces. Careful design of the eyes and multiple degrees of freedom (DoFs) dedicated to controlling the field of vision contribute to rich eye expressions. In these environments, the user assumes the roles of interviewee and interviewer and can safely rehearse initiations and responses. The GIT-VICS Program offers trainees the following benefits: (1) active participation rather than passive observation, (2) a unique training experience, and (3) low cost and high accessibility. By having experience not only performing the role of an interviewee but also evaluating and meta-evaluating the interviewers, we expected that the participants could learn the perspectives of others (i.e., the perspectives of the interviewer and the meta-evaluator). Thus, we considered that our system would be effective in facilitating the acquisition of interview skills by individuals with ASD. This study investigated the effectiveness of the GIT-VICS Program in facilitating skill acquisition for face-to-face interviews.

## 2. Materials and methods

### 2.1. Participants

This study was approved by the Ethics Committee of Kanazawa University. The participants were recruited through flyers explaining the details of the experiment. All procedures performed in studies involving human participants were conducted in accordance with the ethical standards of the institutional and/or national research committee, and the 1964 Declaration of Helsinki and its subsequent amendments. After receiving a full explanation of the study, all participants and their guardians agreed to participate. Written informed consent for the release of any potentially personally identifiable images or data contained in this article was obtained from the individuals and/or legal guardians of the minors. The authors declare that no conflicts of interest exist in this study. The inclusion criteria included (1) a diagnosis of ASD according to the Diagnostic and Statistical Manual of Mental Disorders, Fifth Edition (DSM-5) (1) from an experienced psychiatrist, (2) intelligence quota (IQ) ≥70, (3) age 20–29 years, (4) unemployed and actively seeking employment, and (5) not taking medication. The exclusion criterion for the ASD group was medical conditions associated with ASD (e.g., Shank3, fragile X syndrome, and Rett syndrome). At enrollment, the diagnoses of all participants were confirmed by an experienced psychiatrist with >15 years of experience in ASD using DSM-5 criteria and standardized criteria from the Diagnostic Interview for Social and Communication Disorders (DISCO) (26). The DISCO has been reported to exhibit good psychometric properties (27). All participants had been acquaintances for at least 1 year. A Mini-International Neuropsychiatric Interview (28) was conducted to rule out other psychiatric diagnoses.

The participants completed the Autism Spectrum Quotient-Japanese version (AQ-J) (29) to assess behaviors and symptoms specific to ASD. The AQ-J is a short questionnaire containing five subscales (social skills, attention switching, attention to detail, imagination, and communication). Previous studies of the AQ-J have been replicated across cultures (30) and ages (31, 32). Notably, the AQ is sensitive to broader autism phenotypes. In this study, we did not set a cutoff based on the AQ-J score and used only the DSM-5 and DICSO to diagnose ASD and determine the participants to be included in our study.

Full-scale IQ scores were measured by either the Wechsler Adult Intelligence Scale–Third Edition or the Japanese Adult Reading Test (JART) (33). The latter is a standardized cognitive function test used to estimate the premorbid IQ of individuals with cognitive impairment. The JART is valid with respect to IQ measurements. The JART results were compared to those of the WAIS-III (33).

The severity of each participant’s social anxiety symptoms was measured using the Liebowitz Social Anxiety Scale (LSAS) (34). This clinician-administered scale consists of 24 items: 13 describing performance situations and 11 describing social interactions. Each item was rated separately for “fear” and “avoidance” on a four-point categorical scale. Receiver operating curve analyzes showed that an LSAS score of 30 is correlated with minimal symptoms and is the optimal cutoff value for distinguishing between individuals with and without social anxiety disorders (35).

The ADHD Rating Scale (ADHD-RS) (36) includes 18 items related to inattentive and hyperactive–impulsive symptoms, scored on a four-point scale (0 = never, 1 = sometimes, 2 = often, 3 = very often), and assesses symptom severity over the past week. The total score is calculated as the sum of the scores for all 18 items.

### 2.2. Apparatus

**Figure 1:**
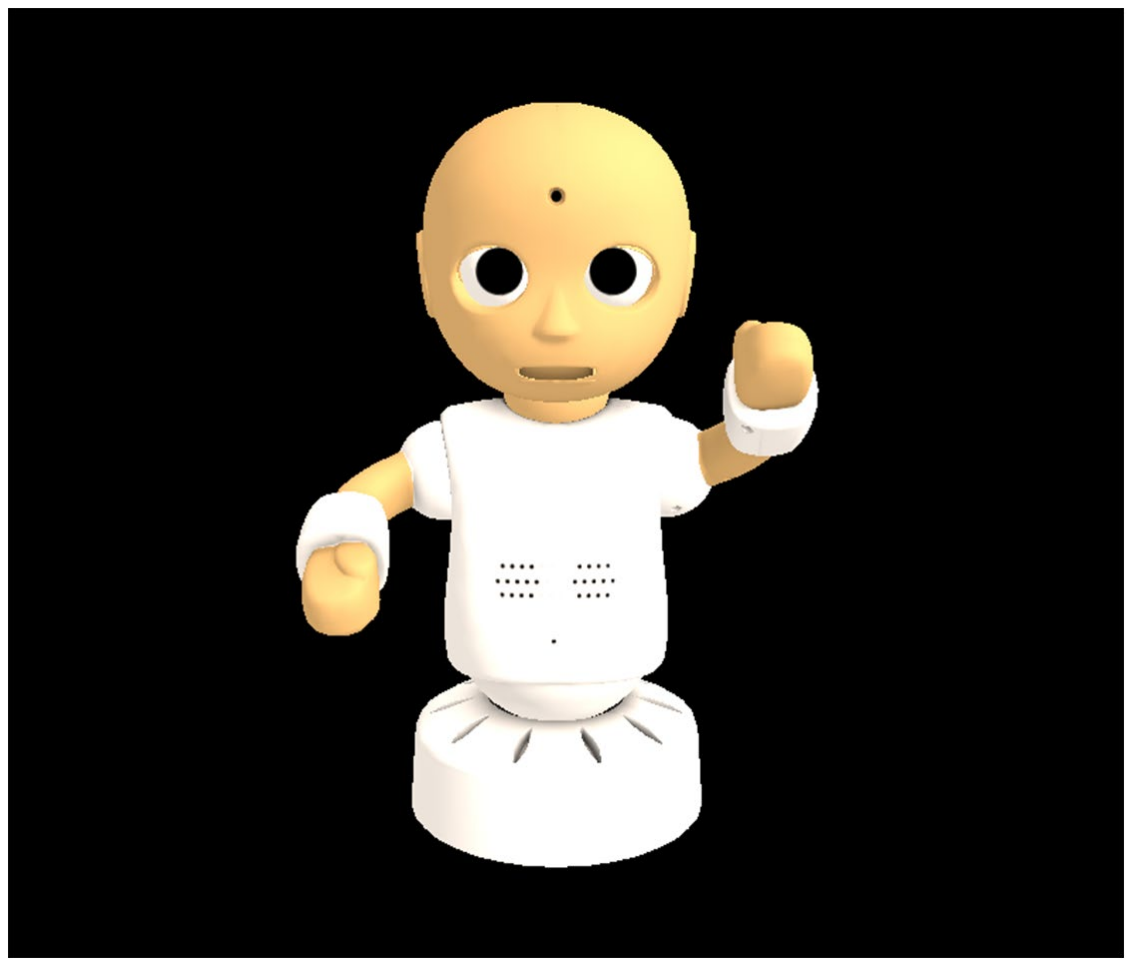
CG CommU.

An online virtual conferencing system was used, with CG robots representing the proxy agents of the participants (Figure 1). A humanoid robot, CommU (Vstone Co., Ltd.), which has been used in several studies on the treatment and education of individuals with ASD (17, 37, 38), used a three-dimensional CG model. The participants could talk to each other as if they became a CommU in the virtual conversation room; therefore, this conferencing system was called CommU-Talk. The CommU had 14 DoFs: waist (2), left shoulder (2), right shoulder (2), neck (3), eyes (3), eyelids (1), and lips (1). The CommU’s face, which contributes to its facial expression by focusing on the design of its eyes and incorporating multiple DoFs dedicated to gaze control, can display a variety of simplified facial expressions that are less complex than those of a real human face. Its small and cute appearance is expected to alleviate the fear of participants with ASD. In this experiment, three CG robots were remotely teleoperated by three participants (one interviewee and two interviewers) and displayed on the screens of their laptops.

In CommU-Talk, the participants’ voices were captured using microphones and replayed. Furthermore, the captured voices were used to automatically produce the nonverbal behaviors of the CG robots so that they looked like they were actively speaking and attentively listening to each other; the speaking CG robot moved its lips and made hand gestures in synchrony with its voice, while the listening robots directed their gazes and nodded toward the speaking robot. The automatic function to produce nonverbal behavior synchronized with voices is expected not only to enable easy control of CG robots but also to provide users with a rich sense of agency and a sense of being attended to by others, independent of the participant’s usual behavior. Furthermore, the user can click the CG robots of the other participants on the screen to make the CG robot produce a gaze movement toward the clicked robot, which is expected to make the user accustomed to consciously looking at the listeners.

**Figure 2:**
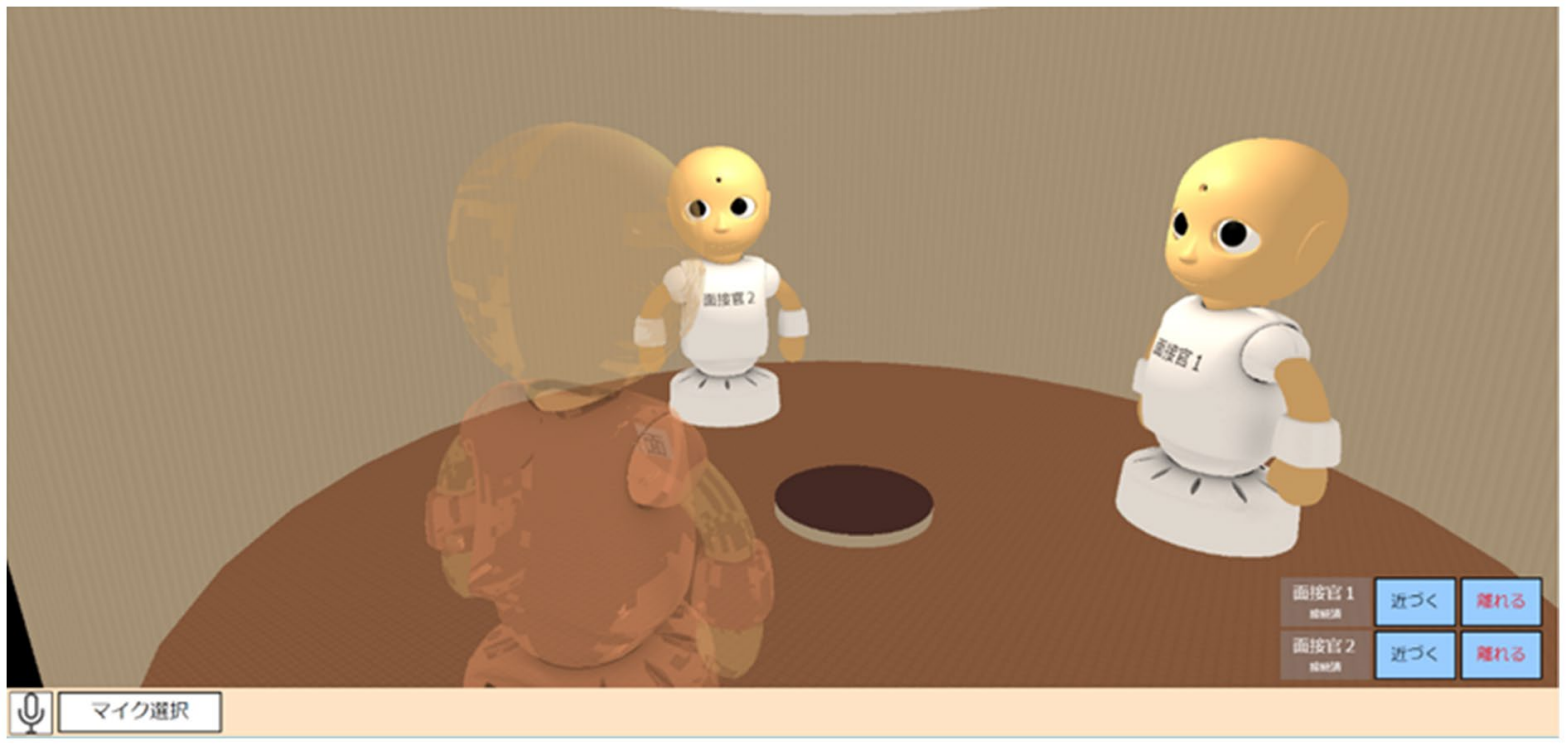
The scene of a mock online job interview using the CG CommU.

On the screen of the interviewee’s laptop computer, the avatar operated by the interviewee showed its back, whereas the other two avatars operated by the interviewers faced each other (Figure 2). On the screen of each interviewer’s laptop computer, an avatar operated by the interviewer displayed its back, while the other two avatars operated by the interviewee and the other interviewer displayed their faces. The same interview scene captured from different angles was recorded and played during the discussion phase using a conventional online conferencing system (Zoom) to share with the meta-evaluators.

### 2.3. Procedures

Fifteen male participants were divided into three groups with five participants each. In each group, they alternately played the roles of interviewer, interviewee, and meta-evaluator in the GIT-VICS Program sessions conducted over 8 or 9 days over 3 consecutive weeks. Fourteen participants used a CG robot to play the role of interviewee five times during the GIT-VICS Program, while one skipped it twice because of an unexpected absence due to family misfortune. In all sessions, two interviewers played the roles of the CG robots and evaluated the interviewees with two meta-evaluators. The number of meta-evaluators was reduced when only one was absent, whereas the session was postponed when more than one was absent. The average times (SD) spent playing the role of interviewee, interviewer, and meta-evaluator were 4.9 (0.5), 9.7 (1.9), and 8.8 (1.5) minutes, respectively.

**Figure 3:**
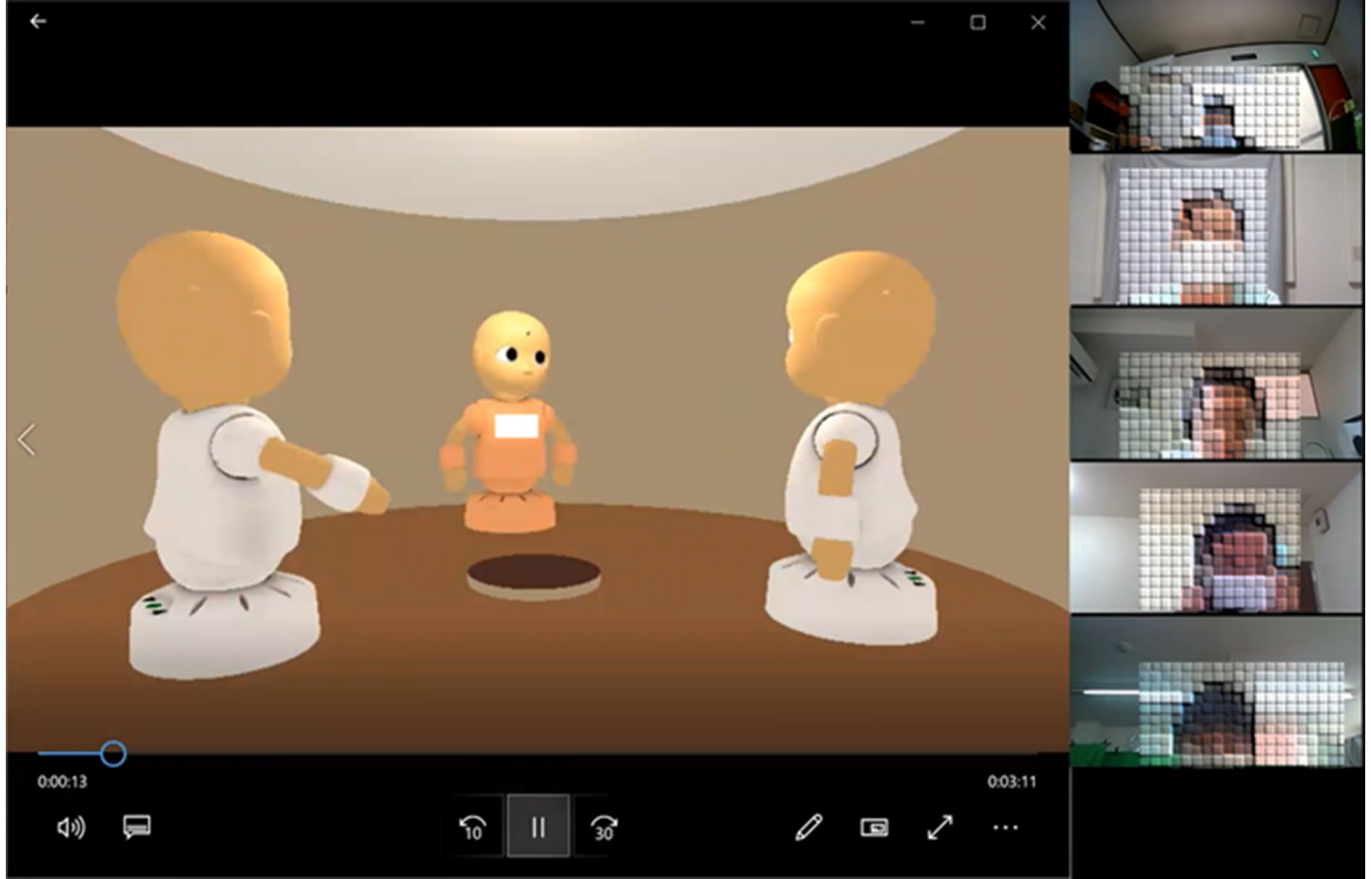
Example of participants of the mock online job interview training.

Each session consisted of four phases: first job interview, feedback, second job interview, and final comments. The first mock online job interview was conducted in CommU-Talk for approximately 3 min, with only two interviewers and one interviewee. Subsequently, the feedback was started in a conventional online meeting system (Zoom), in which not only the participants in the first phase but also the meta-evaluators and experimental assistants participated (Figure 3). The experimental assistant shared the recorded video of the interview in the first phase and facilitated discussions to evaluate the interviewees. The interviewee was then asked to complete a second job interview with the same two interviewers on CommU-Talk. Finally, an additional conversation for the final comments was held in the same online meeting system, and all participants, except for the experimental assistant, provided final short comments on the interviewee’s second performance.

To allow the participants to simulate an interview situation, each interviewee was given fictitious recruitment information containing company names, job types, and working conditions from which they selected one as the company to which to apply. The job types included data entry clerks, supermarket inventories, janitors, restaurant cooking assistants, nursing assistants, and newspaper delivery personnel. Two other participants alternately asked questions based on the prepared lists as interviewers (Supplementary material S1), which the interviewees answered. The interviewees were asked to concentrate on answering the questions in their first and second sessions. After the third session, they were asked to direct the gaze of the robot alternately toward the interviewers by clicking on the interviewers’ CG robots on the screen.

In the feedback phase, all participants talked while showing their faces in Zoom. The experimental assistant shared a video clip capturing the interview scene in CommU-Talk and facilitated a discussion to evaluate the performance of the interviewee based on the prepared scripts (Supplementary material S2). The interviewers and meta-evaluators assessed the performance of the interviewees by completing and discussing a prepared scoring sheet. Therefore, the meta-evaluators evaluated the performances without any direct experience as interviewers. The scoring sheet included evaluation items in terms of verbal factors (appropriate word use, enthusiasm, and appropriate question responses). In addition, a different item on nonverbal factors was included depending on the number of sessions, as the interviewee for the current interviewee to be evaluated. Appropriate speaking speed, vocal fluency, eye contact, response with appropriate timing, and vocal volume were added to the first to fifth sessions, respectively. In the fourth and fifth sessions, an item regarding appropriate eye contact was added. Subsequently, for each item, the interviewers and meta-evaluators explained and discussed the reasons for their scores to determine the score. The interviewee listened to the discussions as observers.

Finally, in the second phase, the interviewees participated in a mock online job interview using CG robots as in the first setup. Each session lasted approximately 50–60 min (3 min for the first and second job interviews, 40 min for feedback, and 8 min for final comments).

**Figure 4:**
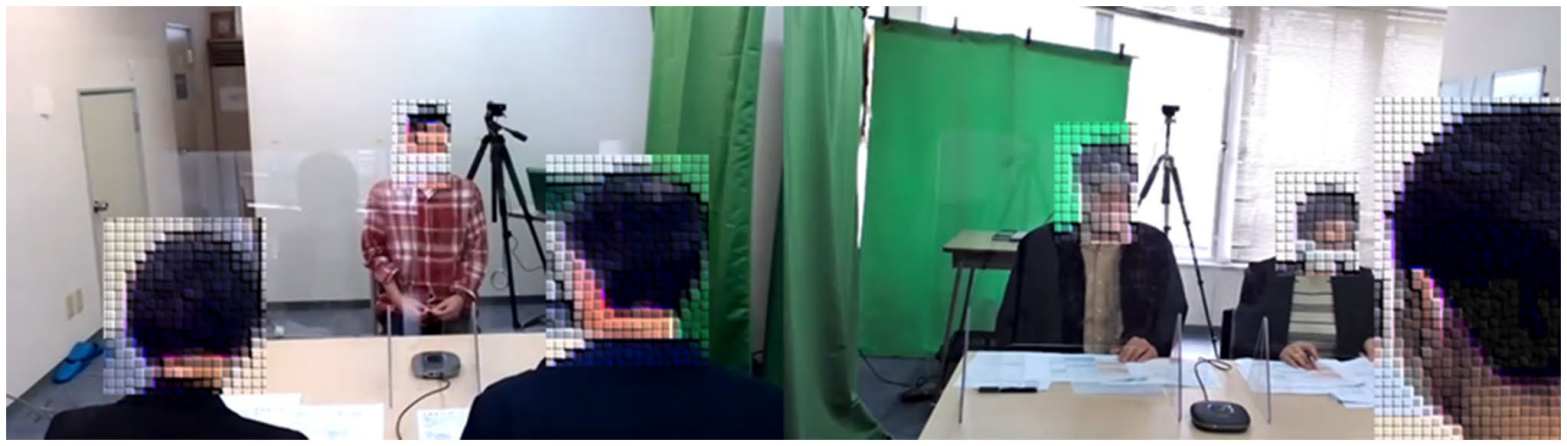
The scene of a mock face-to-face job interview with an experienced human interviewer.

Before and after the GIT-VICS Program, the participants underwent a mock face-to-face job interview with two experienced human interviewers (MFH) (Figure 4) to evaluate its effect.

### 2.4. Self-evaluation

After the first MFH (i.e., before the GIT-VICS Program) and the second MFH (i.e., after the GIT-VICS Program), we asked the participants to complete a questionnaire about their interview performance. The included items surveyed verbal competence (appropriate word use, appropriate question response), nonverbal competence (speaking calmly, being enthusiastic, appropriate speaking speed, appropriate vocal fluency, responding with appropriate timing, and appropriate vocal volume), and interview performance (sounding honest, sounding interested in the job, and being confident in receiving job interviews). These items were rated on a seven-point Likert scale (ranging from “1 = strongly disagree” to “7 = strongly agree”). In addition, to assess the extent to which each participant in the position of interviewee understood that their point of view differed from that of the interviewer or meta-evaluator, we asked them to rate, on a scale of 1–5 (1: I cannot understand the perspectives of the interviewer and meta-evaluators in the job interview setting at all to 5: I can understand the perspectives of the interviewer and human resources in a job interview setting perfectly) the extent to which they understood the point of view of the interviewers and meta-evaluators.

### 2.5. Other’s evaluation

The two raters independently evaluated the interview performances after the first MFH (before the GIT-VICS Program) and the second MFH (after the GIT-VICS Program) by watching video recordings of the sessions. Before the experiment, both raters received approximately 10 h of training in interview scoring while watching videos of interview scenes. The raters scored the interviews using a seven-point Likert scale related to verbal competence (appropriate word use, appropriate question response), nonverbal competence (speaking calmly, being enthusiastic, appropriate speaking speed, appropriate vocal fluency, appropriate timing, appropriate vocal volume, appropriate eye contact, and natural facial expressions), and interview performance (sounding honest and sounding interested in the job). The ratings ranged from 1 (very poor) to 7 (very excellent).

The raters also evaluated whether the participants looked at the interlocutor’s face every 5 s in the MFH. They calculated the number of frames in the interview to determine whether the interviewee looked at the interlocutor’s face when the interviewee was being asked questions. The raters also calculated the number of frames in the interview to determine whether the interviewees looked at the interviewer’s face when they responded to the interview questions.

Both raters were blinded to the day of the shooting (i.e., the first or second MFH). The primary and secondary raters showed moderate reliability [intraclass correlation coefficient (ICC) = 0.64] in interview performance scores. The primary secondary rater showed substantial reliability [ICC = 0.89] regarding whether the interviewee looked at the interviewer’s face. The primary rater’s score was used in this study because he was a more experienced evaluator than the second rater. After the intervention, the participants’ supporters, trainers, and job coaches were asked the following question: “Did the participants learn to understand the point of view of the interviewer after the intervention?”

### 2.6. Statistical analysis

Statistical analyzes were performed using IBM SPSS Statistics for Windows, version 24.0 (IBM Corp., Armonk, NY, United States). Descriptive statistics were calculated for the samples.

To assess the degree of improvement in self-evaluation (appropriate word use, appropriate question response, speaking calmly, being enthusiastic, appropriate speaking speed, appropriate vocal fluency, responding with appropriate timing, appropriate vocal volume, sounding honest, sounding interested in the job, being confident in receiving job interview, and understanding the perspectives of interviewers and meta-evaluators in job interview setting) and evaluation of others (appropriate word use, appropriate question response, speaking calmly, being enthusiastic, appropriate speaking speed, appropriate vocal fluency, responding with appropriate timing, appropriate vocal volume appropriate, eye contact, natural facial expressions, sounding honest, sounding interested in the job, recognition of the point of view of interviewers and meta-evaluators, and looking at the interlocutor’s face when being asked questions by the interviewer and when the interviewee is responding to the interview’s questions) between the first and second MFH, a paired *t*-test was performed. We employed an alpha level of 0.05 for these analyzes.

## 3. Results

### 3.1. Demographic data

Fifteen individuals with ASD participated in this study. One participant dropped out of the experiment before the second MFH because of family misfortune (i.e., he could finish the first MFH or the entire course of the GIT-VICS Program). Fourteen participants completed the trial procedure without any technical challenges or participant distress that would have led to the termination of the session (see Table 1 for the experimental details of the participants who could finish). The participants’ performances were carefully monitored to ensure that all participants were focused during the trial and remained highly motivated from the beginning to the end of the experiment.

**Table 1:** Descriptive statistics of participants.

Characteristics	*n* = 14 *M* (SD)
Age (years)	23.3 (2.7)
Gender (Male: Female)	13:1
Full scale IQ	94.4 (11.5)
AQ-J	29.3 (4.5)
LSAS-J	55.0 (27.3)
ADHD-RS	14.7 (4.7)

M, mean; SD, standard deviation.

### 3.2. Main result

The self-evaluation showed significant increases between the first and second MFH in appropriate question response (3.47 vs. 4.73; *p* = 0.005), speaking calmly (3.67 vs. 4.73; *p* = 0.027), and sounding interested in the job (3.79 vs. 5.00; *p* = 0.003). The recognition of the importance of the perspectives of interviewers and meta-evaluators also increased significantly after the second MOH compared to after the first MOH (3.50 vs. 1.79; *p* < 0.001; Table 2).

**Table 2:** Means and standard error of the mean of self-evaluation at first MFH and second MFH.

Outcome	First MFH *M* (SD)	Second MFH M (SD)	*t*	Statistics *F*	*p*
Verbal competence					
Appropriate word use	3.93 (2.09)	4.60 (1.30)	−1.404	13	0.182
Appropriate question response	3.47 (1.60)	4.73 (1.49)	−3.300	13	0.005^**^
Nonverbal competence					
Speaking calmly	3.67 (1.84)	4.73 (1.58)	−2.477	13	0.027^*^
Being enthusiastic	3.87 (1.69)	4.33 (1.40)	−0.940	13	0.363
Appropriate speaking speed	3.87 (2.13)	4.53 (1.55)	−1.673	13	0.116
Appropriate vocal fluency	3.60 (1.72)	3.87 (1.69)	−0.576	13	0.573
Respond with appropriate timing	3.71 (1.90)	4.29 (1.77)	−1.295	13	0.218
Appropriate vocal volume	3.79 (2.16)	4.57 (1.60)	−1.712	13	0.111
Interview performance score					
Sounding honest	4.57 (2.10)	4.79 (1.85)	−0.434	13	0.671
Sounding interested in the job	3.79 (1.81)	5.00 (1.52)	−3.631	13	0.003^**^
Being confident in receiving job interview	3.36 (1.99)	4.36 (1.55)	−1.803	13	0.095
Understanding the importance of the perspectives of interviewers and human resources	1.79 (0.89)	3.50 (0.65)	−6.000	13	<0.001^***^

M, mean; SD, standard deviation. MFH: a mock face-to-face job interview with a professional human interviewer. *p < 0.05, **p < 0.01, ***p < 0.001.

The evaluation of others showed significant increases between the first and second MFH in appropriate word use (3.86 vs. 5.00, *p* = 0.017), appropriate question response (3.43 vs. 4.86, *p* = 0.001), speaking calmly (3.86 vs. 4.71, *p* = 0.005), being enthusiastic (3.86 vs. 5.14, *p* < 0.001), appropriate vocal fluency (3.43 vs. 4.00, *p* = 0.006), responding with appropriate timing (3.71 vs. 4.86, *p* < 0.001), appropriate vocal volume (4.07 vs. 5.00, *p* = 0.004), appropriate eye contact (3.00 vs. 4.29, *p* = 0.002), natural facial expressions (3.50 vs. 4.43, *p* = 0.004), and sounding interested in the job (4.43 vs. 5.50, *p* = 0.008; Table 3).

**Table 3:** Means and standard error of the mean of other’s evaluation at first MFH and second MFH.

Outcome	First MOH *M* (SD)	Second MOH *M* (SD)	*t*	Statistics *F*	*p*
Verbal competence					
Appropriate word use	3.86 (1.35)	5.00 (0.87)	−2.738	13	0.017^*^
Appropriate question response	4.14 (0.86)	4.36 (1.08)	−4.163	13	0.001^**^
Nonverbal competence					
Speaking calmly	3.86 (1.29)	4.71 (0.83)	−3.379	13	0.005^*^
Being enthusiastic	3.86 (1.10)	5.14 (0.77)	−7.870	13	<0.001^***^
Appropriate speaking speed	4.14 (0.86)	4.36 (1.08)	−1.147	13	0.272
Appropriate vocal fluency	3.43 (1.09)	4.00 (1.03)	−3.309	13	0.006^**^
Respond with appropriate timing	3.71 (1.33)	4.86 (1.23)	−4.947	13	<0.001^***^
Appropriate vocal volume	4.07 (0.92)	5.00 (0.68)	−3.484	13	0.004^**^
Appropriate eye contact	3.00 (1.41)	4.29 (0.99)	−3.994	13	0.002^**^
Natural facial expression	3.50 (0.94)	4.43 (0.65)	−3.484	13	0.004^**^
Interview performance score					
Sounding honest	5.71 (0.61)	6.07 (0.27)	−1.794	13	0.096
Sounding interested in the job	4.43 (1.70)	5.50 (1.02)	−3.160	13	0.008^**^

M, mean; SD, standard deviation. MFH, a mock face-to-face job interview with a professional human interviewer. *p < 0.05, *p < 0.01, **p < 0.001.

We could not evaluate whether one participant looked at the interlocutor’s face because part of the video of the first MFH was blurry. Thus, we evaluated whether 13 of the participants looked at the interviewer’s face. We observed a significant increase between the first and second MFH when looking at the interviewer’s face when the interviewees responded to questions (51.9 vs. 66.5, *p* = 0.029) but not in looking at the interviewer’s face when asked questions (53.0 vs. 62.0%; *p* = 0.166; Table 4).

**Table 4:** Means and standard error of the mean of the extent of participant look at the interviewer’s face at first MFH and second MFH.

Outcome	First MOH *M* (SD)	Second MOH *M* (SD)	*t*	Statistics *F*	*p*
Looking at the interviewer’s face when being asked questions by the interviewer	51.9 (29.0)	66.5 (25.6)	−2.471	12	0.029^*^
Looking at the interviewer’s face when the interviewee is responding to the interview’s questions	53.0 (33.0)	62.0 (31.8)	−1.476	12	0.166

*M*, mean; SD, standard deviation. MFH, a mock face-to-face job interview with a professional human interviewer. *p < 0.05.

In the semi-structured interview, the participants’ supporters responded to the following prompt: “All students seemed to learn to better understand the point of view of the interviewer.” At the 1-year follow-up after the intervention, nine participants had passed job interview tests and gained employment. In our interview, 1 year after the intervention, all participants answered, “The experience with the GIT-VICS Program was the trigger to put ourselves in the interviewer’s shoes.”

## 4. Discussion

The current study evaluated the feasibility of the GIT-VICS Program, a new group-based online job interview training program that uses CG robots. The completion rate suggested that participants who received the GIT-VICS Program continued to participate without losing motivation. Using a CG robot and learning the importance of interview skills by experiencing other perspectives (i.e., the viewpoints of the interviewer and meta-evaluators) may have sustained their motivation. The GIT-VICS Program contributed to improvements in various job interview skills (i.e., verbal competence, nonverbal competence, and interview performance). These results occurred in the absence of specific interview skill training by professionals (i.e., the improvements were based only on practice and feedback from participants with ASD).

In this program, playing the role of an interviewer using CG CommU had many advantages over conversing face-to-face in an online setting. Sensory overstimulation from humans in face-to-face conversations is a serious problem for individuals with ASD and interferes with the processing of social signals (39). By using this system in the present program, interviewers and meta-evaluators could not see the actual appearance of the interviewees. Therefore, the interviewees were free from the awareness of being watched by others, which may be linked to decreased interpersonal anxiety in online mock job interview training. Furthermore, a growing body of literature indicates that many individuals with ASD have the desire and motivation to use technology (40); thus, the technology behind the CG CommU might increase users’ enthusiasm and focus on the program.

The participants in this study experienced improvements in some self-evaluation items (appropriate question responses, speaking calmly, sounding interested in the job, and recognizing the importance of the perspectives of interviewers and meta-evaluators) but did not experience improved self-confidence in their self-evaluations. In the GIT-VICS Program, only individuals with ASD participated in the feedback phases; they identified the weak points of the interviewees without paying due respect, which may be linked to the fact that they could not improve their self-confidence. Evidence indicates that the more confident one is in performing an interview, the greater the social engagement during the interview, and the more effective the verbal and nonverbal communication strategies (41, 42). Future projects should be modified so that participants can point out both the weaknesses and strengths of the interviewees and ensure that they point out the weaknesses.

The participants received training only in an online setting and not in a face-to-face job interview setting. In addition, we used a simple humanoid robot, the CG CommU, which is difficult to generalize to humans. As individuals with ASD have low self-esteem (43) and many are not good at generalization (44), they may still not have confidence in the face-to-face job interview setting after the GIT-VICS Program.

The participants in this study could take the viewpoints of not only an interviewer evaluating the interviewee but also a meta-evaluator who argues for the validity of the evaluations by interviewers and another meta-evaluator. These experiences might deepen the participants’ understanding of the perspective of the interviewer in job interview situations–that is, how they would be evaluated and what they should do to be evaluated highly.

The participants showed improvements in most items (appropriate word use, appropriate question response, speaking calmly, being enthusiastic, appropriate vocal fluency, responding with appropriate timing, appropriate vocal volume, eye contact, natural facial expressions, sounding interested in the job, recognition of the importance of the point of view of interviewers and meta-evaluators, and looking at the interlocutor’s face when asked questions by the interviewer and when the interviewee was responding to the interview questions) in the evaluation of others. Completion of the GIT-VICS Program increased the participants’ recognition of the importance of the perspectives of interviewers and meta-evaluators, concentrating during the trials, and being highly motivated from the start to the end of the program, which may have contributed to the improvements in most items. As many individuals with ASD are poor at generalization (44), improving the evaluation of others in a real-world job interview setting after the GIT-VICS Program is significant.

The results of this study showed that the GIT-VICS Program did not improve looking at the interlocutor’s face when the interviewee listened to the interviewers’ utterances but did so when he or she spoke to them. These different effects may be explained by how the participants experienced looking in the GIT-VICS Program. In the GIT-VICS Program, when the interviewer listened to the utterances of the interviewers’ CG robots, the interviewee’s CG robot automatically looked toward the speaking CG robot. However, when the interviewer spoke to answer questions, the interviewer was also asked to consciously control the gaze of his CG robot by clicking on the target CG robot to be looked at in the last three trials. Moreover, the looking performance was explicitly evaluated in the feedback session. In other words, the participants only became accustomed to the active experience, which could be viewed as an improvement in looking at the interlocutors’ faces when answering.

This study has several limitations. First, the sample size was relatively small. In addition, most participants were male. Future studies involving larger samples of female participants are required to provide more meaningful data regarding the potential use of this system. Second, this study was not a controlled study. There was no sham training group to compare with the online CG robot training group. At the time that this experiment was conducted, the Japanese government had declared a state of emergency due to the proliferation of coronavirus disease 2019 (COVID-19), so we could not ask participants to participate in a controlled setting. Given the urgent need for individuals with ASD to prepare for online job interviews, a no-control pilot study had to be conducted.

Nonetheless, it is worthwhile to report the results in their current form, which demonstrated improvements in areas in which the intervention was challenging, as this underscored the need for a large-scale follow-up study with a control group to establish the efficacy of the intervention. The ultimate goal of the program is to improve the participants’ communication skills in everyday life and give them a competitive edge when seeking employment or volunteer positions. An employment support facility is required to ensure that our system works. Given the high cost of caring for individuals with ASD (45), it is of critical economic importance to help these individuals achieve competitive status. Future long-term longitudinal studies of work support facilities are needed to test whether this program can achieve this goal.

This is the first study to evaluate the effect of mock online job interview training using CG robots in increasing the ability of individuals with ASD to participate in face-to-face job interviews. Our program improved most interview performance items related to the evaluation of others in individuals with ASD. This result occurred in the absence of specific interview skill training by professionals. If professionals can provide appropriate guidance, individuals with ASD may improve on other items. Given the promising results of this study and to draw clear conclusions about the efficacy of CG robots for mock online job interview training, future studies adding appropriate guidance for manner of job interview by experts are needed.

## Data availability statement

The raw data supporting the conclusions of this article will be made available by the authors, without undue reservation.

## Ethics statement

The studies involving human participants were reviewed and approved by Ethics Committee of Kanazawa University. The patients/participants provided their written informed consent to participate in this study.

## Author contributions

YY and HK designed the study, conducted the experiments, conducted the statistical analyzes, analyzed and interpreted the data, and drafted the manuscript. YY, TM, KS, HI, MM, HH, and AK conceptualized the study, participated in its design, assisted with data collection and scoring of behavioral measures, analyzed and interpreted the data, drafted the manuscript, and critically revised the manuscript for important intellectual content. HK approved the final version to be published. All the authors have read and approved the final version of the manuscript.

## Funding

This work was supported by JST and Moonshot R&D, Grant Number JPMJMS2011.

## Conflict of interest

The authors declare that the research was conducted in the absence of any commercial or financial relationships that could be construed as a potential conflict of interest.

## Publisher’s note

All claims expressed in this article are solely those of the authors and do not necessarily represent those of their affiliated organizations, or those of the publisher, the editors and the reviewers. Any product that may be evaluated in this article, or claim that may be made by its manufacturer, is not guaranteed or endorsed by the publisher.
