# Supplementary material for: A new group-based online job interview training program using computer graphics robots for individuals with autism spectrum disorders
Source: Front Psychiatry. 2023 Jul 3;14:1198433. doi: 10.3389/fpsyt.2023.1198433 (PMC10350627; doi:10.3389/fpsyt.2023.1198433)
Supplement: Supplementary file 1 [file Data_Sheet_1.DOCX]

**Supplementary Material 1**

The dialog between the interviewee and interviewers in the first and second job interview phases of the GIT-VICS Program was partially structured by using the script presented below. The sentences listed in (1), (2), and (3) were the utterances of the interviewer. Two interviewers uttered a sentence from list (1) alternately, waited for a response from the participant, and then responded with a sentence from list (2). The utterances in list (3) were sometimes inserted while waiting for the participant to respond to develop the theme more deeply. The sentences were presented in the numerical order shown in list (1) on all days of the experiment, while one of the two sentences was chosen from list (3) for use each day of the experiment.

1. Examples of scripts for the first and second online job interview phases
2. Please take a seat.
3. Good afternoon. Hello there.
4. Thank you for applying to my company, XX.
5. Well then, could you introduce yourself?
6. Would you please tell me the reason for your application?
7. Why do you want this job? Please tell me.
8. What are the things you are good at?

What is your special talent?

1. What are the things you are not good at?
2. How do you adjust for the things you are weak in?
3. What kind of things would you like to do in our company?

Please tell me what you can do.

1. What are you doing in vocational training school?
2. Have you ever failed at your work?
3. Please explain your disability briefly.
4. How are you feeling now?
5. In my company, we have many jobs that require standing. Is that all right?
6. Are you able to carry heavy things?
7. Are you on any medicine now?
8. Is there anything you would like us to consider?
9. Which route did you take to come here from your home?
10. Are there any specific working days or hours you want?
11. Is there anything else you would like to tell us?
12. I understand. Thank you very much.
13. Words and phrases used as responses (to facilitate the conversation)

・ Yes

・ Certainly

・ All right

・ Very well

・ Thank you

・ I see. Now I understand.

・ Oh, I see. I got it.

・ I see. That makes sense.

・ Great!

・ Wonderful!

・ Fantastic!

・ Incredible!

1. Phrases used to elicit further response

・ Please tell me a bit more in detail.

・ Why do you think so?
