# Supplementary material for: A new group-based online job interview training program using computer graphics robots for individuals with autism spectrum disorders
Source: Front Psychiatry. 2023 Jul 3;14:1198433. doi: 10.3389/fpsyt.2023.1198433 (PMC10350627; doi:10.3389/fpsyt.2023.1198433)
Supplement: Supplementary file 2 [file Data_Sheet_2.DOCX]

**Supplementary Material 2**

The dialog between the meta-evaluators and other participants (i.e., interviewee and interviewers) in the feedback phase was partially structured by using the script presented below. The sentences listed in (1) were the utterances of the meta-evaluators. Two meta-evaluators uttered a sentence from list (1) alternately and waited for a response from the participant.

Examples of scripts for the feedback phase

1. We have a conference in the feedback phase. Mr./Mrs. A (i.e., interviewee), please take notes and listen. At the end of the session, you will be asked to report what you have learned.

2. Dear interviewers, about the item “○○”, please state the score, the reason, and the scene explaining the reason.

3. If you have any objections to the current assessment, please let me know.

4. Mr./Mrs. A, what are three things that you will keep in mind that you learned in this conference?

5. Let’s start mock job interview training again by being aware of the guidance you have received now.
